# Fetal growth and incidence of atopic dermatitis in early childhood: Results of the Ulm SPATZ Health Study

**DOI:** 10.1038/s41598-018-26440-2

**Published:** 2018-05-23

**Authors:** Chad A. Logan, Johannes M. Weiss, Frank Reister, Dietrich Rothenbacher, Jon Genuneit

**Affiliations:** 1 Institute of Epidemiology and Medical Biometry, Ulm University, Ulm, Germany; 2 Department of Dermatology and Allergic Diseases, University Medical Center Ulm, Ulm, Germany; 3 Department of Gynecology and Obstetrics, University Medical Center Ulm, Ulm, Germany; 4 Member of ‘In-FLAME’ the International Inflammation Network, World Universities Network (WUN), Leeds, UK

## Abstract

Fetal growth may be a precursory factor in observed association between birthweight and atopic dermatitis (AD), however, recent studies utilizing fetal ultrasound-based data have reported contradictory results. This study aims to clarify previous findings through comprehensive investigation of association between several trimester-specific ultrasound-based anthropometric measures with AD diagnosis by age 3 years. Measurements of 386 newborns in the Ulm SPATZ Health Study were converted into adjusted z-scores categorized as “low” (≤1 SD below mean), “normal,” or “high” (≥1 SD above mean). AD cases were defined using parent- or pediatrician-report of physician-diagnosis or clinical diagnosis. Adjusted risk ratios (RR) with 95% confidence intervals (95% CI) were calculated using modified Poisson regression. Compared to normal, both low and high 2^nd^ trimester abdominal circumference [RR 1.51, (95% CI 1.01; 2.24) and 1.83 (1.21; 2.76)], high 2^nd^ trimester head- abdominal circumference ratio [1.69 (1.16; 2.48)], and faltering 2^nd^ to 3^rd^ trimester [1.59 (1.04; 2.43)] head circumference were associated with greater AD risk. High 3^rd^ trimester femur length [0.54 (0.31; 0.94)] was associated with lower risk. Using more inclusive exposure cut-points (0.8 SD), lower 1^st^ trimester crown-rump length was also associated with greater AD risk. Our data suggest several different patterns of fetal growth may be differentially associated with AD.

## Introduction

Several studies have reported cross-sectional associations between birthweight, neonatal adiposity, or other anthropometric measures at birth and increased risk of atopic dermatitis (AD) beginning in infancy or early childhood^1–5^. Though these results suggest that overall fetal growth may be a risk factor for AD, studies investigating fetal growth throughout gestation may offer more detailed data for causative research on potential determinants of this, and potentially other, atopic diseases^6–9^.

Though few previous studies have directly investigated associations between fetal growth using ultrasound-based anthropometric measurements and AD or other atopic outcomes^10–14^, several potentially important but inconsistent results have been reported. Greater 1^st^ to 2^nd^ trimester ratio of crown-rump length to biparietal diameter and 2^nd^ to 3^rd^ trimester abdominal circumference have been separately reported to be associated with increased risk of parent-reported physician diagnosis of AD by age 10 years^11^ and by age 4 years^12^ respectively. In contrast to the latter finding, a smaller but more recent study reported accelerated 2^nd^ to 3^rd^ trimester abdominal circumference was associated with decreased risk of parent-reported physician diagnosis of AD by age 2 years^14^. Another study observed that greater 2^nd^ trimester head circumference may be associated with decreased odds of parent-reported physician diagnosis of AD by age 5 years^13^. Finally, it should also be noted that several of the studies mentioned also reported null results which contrast with some of these statistically significant findings.

Inconsistencies between results may be attributable to several factors including differences in study populations, gestational age and anthropometric calculations, antenatal assessment periods and exposure definitions, AD case definition and inconsistencies related to parent reporting of AD diagnosis, data quality, statistical methodology, or a combination of these and other factors^6,9,14^. In particular, linear analysis of non-linear growth patterns typical during certain periods of gestation may have resulted in differential or null findings in some studies but not others.

The primary aim of this analysis was to determine whether fetal anthropometric measurements obtained via ultrasound during the 1^st^, 2^nd^, and 3^rd^ trimester of pregnancy or 2^nd^ to 3^rd^ trimester growth trajectory were associated with physician diagnosis of AD by 3 years of age. To clarify and expand on results from previous studies and provide a more consistent basis for future research, we analyzed several anthropometric measurements cross-sectionally and longitudinally comparable to those reported piecemeal in previous literature. To further improve upon previous studies, we comprehensively investigated the effect of potentially important non-linear fetal growth. Finally, to reduce potential bias commonly encountered in observational studies of childhood AD, we implemented an inclusive case definition based on both parent-reported and pediatrician-reported physician diagnosed AD supplemented by clinical examination.

## Methods

### Study design and population

The Ulm SPATZ Health Study cohort consists of 1006 live newborns of 970 mothers (49% of all 1999 eligible families) recruited from the general population shortly after delivery in the University Medical Center Ulm, Southern Germany, from April 2012 until May 2013. Further details pertaining to study methodology and data collection are available elsewhere^15,16^. In brief, exclusion criteria were outpatient delivery, maternal age <18 years, transfer of the newborn or the mother to intensive care immediately after delivery, and/or insufficient knowledge of the German language. Ethical approval was obtained from and all study protocols were carried out in accordance with guidelines approved by the ethics board of Ulm University (no. 311/11). Participation was voluntary and written informed consent obtained in each case.

### Fetal anthropometrics

Anthropometric measurements were obtained from data recorded during routine obstetric screenings regularly conducted in Germany at 9–12 weeks (1^st^ trimester), 19–22 weeks (2^nd^ trimester), and 29–32 weeks (3^rd^ trimester) gestation. From these records, data were obtained for gestational age at measure, 1^st^ trimester crown-rump length (CRL) and 2^nd^ and 3^rd^ trimester head circumference (HC), abdominal circumference (AC), and femur length (FL). In cases where AC or HC was not directly documented (22% for AC in 2^nd^ and 3^rd^ trimester each; 42% for HC in 2^nd^ trimester and 43% in 3^rd^ trimester), measures were computed using the Ramanujan formula for calculating the circumference of an ellipse and using documented abdominal anteroposterior and transverse diameters and biparietal and occipitofrontal diameters, respectively^17^. In addition to these computations, trimester-specific HC to AC ratio (HC:AC) and estimated fetal weight (EFW) were calculated using the Hadlock 3 formula [Log10EFW=1.326–0.00326*AC*FL + 0.0107*HC + 0.0438*AC + 0.158*FL]^18,19^. No statistically significant differences in z-scores were observed between documented and calculated HC or AC among those with full documentation of both circumferences and diameters (see Supplement Fig. 1).

As obstetricians frequently updated gestational age estimates after 1^st^ trimester screening, more accurate later gestational age estimates were given priority over potentially inaccurate initial estimates in our analyses. To preserve comparability between measurements, ultrasound data reported >21 days from median gestational age calculated for all trimester-specific screenings were not used. Further accounting for gestational age at measure; CRL, HC, AC, FL, HC:AC, and EFW results were converted into z-scores obtained from linear regression models of the effect of gestational age on the respective measure and stratified by child’s gender. Models used to derive z-scores for HC and AC were further adjusted for whether they were obtained directly from written documentation or calculated. Z-scores were then used to categorize measures into “low” (≤1 SD below mean), “normal,” or “high” (≥1 SD above mean) fetal growth.

### AD diagnosis

Children whose parent or pediatrician reported physician AD diagnosis within the 12 months preceding the 1, 2 or 3 year questionnaire-based follow-up period were considered AD cases. In addition, as part of the study protocol, children with potential AD symptoms were invited to undergo dermatological examinations at 6 months (n = 55 attended), 1 year (n = 99 attended), and/or 2 years (n = 60 attended) of age. Children with confirmed dermatological diagnosis during any exam period were considered cases regardless of parent or pediatrician reports. Non-cases were the remaining children with at least one negative parent and pediatrician report within the same or separate time periods. To assess the effect of potential misclassification with the AD case group, disease severity^20^ was assessed with Patient-Oriented Eczema Measure (POEM) scores during the 3 year follow-up period^21^.

### Maternal and birth-related factors

Maternal demographic and lifestyle data including age at delivery, nationality, pre-pregnancy BMI, duration of school education (<12 years or ≥12 years), parity (first birth or greater), history of smoking (within the year prior to pregnancy), and reported physician-diagnosed childhood AD up to 18 years of age were collected using a self-administered standardized questionnaire during the hospital stay following delivery. Clinical data related to the child’s delivery including gender, birthweight, and delivery mode were obtained from electronic hospital records.

### Statistical analyses

Primary analyses were restricted to children with complete 1^st^, 2^nd^, and 3^rd^ trimester fetal growth data and AD outcome. Potential selection bias due to missing data and restrictions was assessed by comparing demographic characteristics (proportions or means) within the analysis subsample to 95% confidence intervals for each characteristic within the full cohort. Modified Poisson regression models^22^ were used to compute risk ratios (RR) for “low” and “high” trimester-specific anthropometric values with AD using the “normal” category as a reference group. Maternal and birth-related factors described above were tested for use as potential covariates in adjusted models. As none of these variables produced a 10% change in point estimate for bivariate association between any fetal growth measure and any AD outcome definition, adjusted models included only the a-priori selected potential confounders child’s gender and maternal education, parity, smoking, and AD history (diagnosis by 18 years of age). The same modelling method was used to calculate crude RR for 2^nd^ to 3^rd^ trimester change from “normal” to “low” (faltering growth) and from “normal” to “high” (accelerated growth) anthropometric category with AD. Here, due to restriction to 2^nd^ trimester “normal”, sample sizes were too low to attempt meaningful adjustment for potential confounders. As in a larger previous study^12^, confidence intervals for model results were not adjusted for multiple comparisons due to the exploratory nature of the investigation and high correlation observed between exposure measures. Finally, sensitivity analyses including removal of subjects with fetal measurements viewed as potential outliers (+/−3 SD from their respective mean), inclusion of subjects with incomplete fetal growth data, evaluation of less stringent cutoff values (0.8 SD and 0.66 SD) used to categorize “low” and “high” anthropometric categories, and separate modelling for association of trimester-specific growth with parent- or pediatrician-reported physician diagnosed AD were conducted. All statistical analyses were performed using SAS 9.4 (SAS Institute, Cary, NC).

## Results

For the purposes of this analysis, the study population was restricted to mothers of singleton newborns (n = 934). Of these, ultrasound data could not be obtained for 69 children and a further 351 children were missing complete data (1^st^ trimester CRL or 2^nd^ or 3^rd^ trimester HC or AC) at one or more trimesters. Among the 514 remaining children, 80 were lost to follow-up before the first dermatological assessment and 48 children were missing parent (n = 2), pediatrician (n = 25), or both (n = 21) reports at all time periods. In total, 386 children were included in our final analyses. Within this group, AD diagnosis was reported by either the parent or pediatrician for 61 children during the year 1 assessment, an additional 32 children in the year 2 assessment, and a further 17 children in the year 3 assessment.

Compared to the full cohort, mothers in this analysis sub-population were slightly older at delivery, more often higher educated, and had lower prevalence of smoking (Table 1). Children were of slightly higher birth weight and less likely to have been delivered via cesarean section. These differences were likely attributable to factors associated with higher participation-related factors among mothers and restriction to singleton deliveries. Gestational age at ultrasound measure and prevalence of AD diagnosis in the analysis sub-population were comparable to those observed in the full cohort.

**Table 1:** Comparison of maternal and child characteristics of the analysis sub-population to the all SPATZ cohort subjects.

Characteristic	SPATZ cohort (N = 1006)			Analysis sub-population (n = 386)	
	N	% or mean	95% CI	n	% or mean
MOTHER					
Age at delivery (years)	1006	32.7	(32.4; 33.0)	386	33.5
Education					
>=12 years	577	58.5%	(55.4%; 61.5%)	257	67.1%
<12 years	410	41.5%	(38.5%; 44.6%)	126	32.9%
Parity					
0 births	547	54.4%	(51.3%; 57.5%)	216	56.0%
>=1 birth	458	45.6%	(42.5%; 48.7%)	170	44.0%
History of Smoking^*^					
No	730	73.4%	(70.6%; 76.1%)	300	77.7%
Yes	265	26.6%	(23.9%; 29.4%)	86	22.3%
Reported having AD in childhood^†^					
No	993	92.1%	(90.5%; 93.7%)	356	92.2%
Yes	85	7.9%	(6.3%; 9.5%)	30	7.8%
CHILD					
Gender					
Male	523	52.0%	(48.9%; 55.1%)	196	50.8%
Female	483	48.0%	(44.9%; 51.1%)	190	49.2%
Birth weight (g)	1005	3278.2	(3245.2; 3311.3)	386	3360.6
Delivery mode					
Vaginal	724	72.0%	(69.3%; 74.8%)	294	76.2%
Cesarean	281	28.0%	(25.2%; 30.7%)	92	23.8%
Gestational age at ultrasound measure (weeks)					
1^st^ trimester	823	10.6	(10.5; 10.7)	386	10.6
2^nd^ trimester	741	20.3	(20.2; 20.4)	386	20.4
3^rd^ trimester	785	30.0	(29.9; 30.1)	386	30.0
AD diagnosis by 3 years					
No	539	73.2%	(70.0%; 76.4%)	276	71.5%
Yes	197	26.8%	(23.6%; 30.0%)	110	28.5%

^*^Report of maternal smoking within year before pregnancy. ^†^Report of a physician’s diagnosis of AD up to age 18 years.

**Figure 1:**
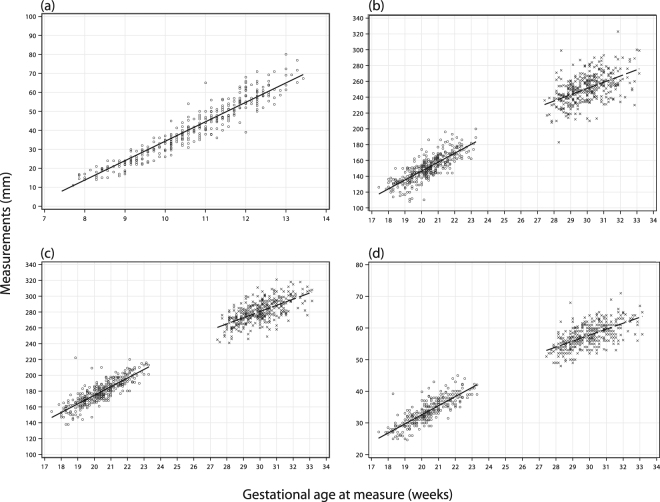
Crown-rump length (**a**), abdominal circumference (**b**), head circumference (**c**), and femur length (**d**) measurements among SPATZ subjects included in the analysis. Note differing x- and y-axis scaling between panels. Circle = 2^nd^ trimester; X = 3^rd^ trimester.

Scatter plots with regression lines for all primary fetal anthropometric measurements by gestational age are available in Fig. 1. As expected, we observed linear increases for individual growth measures with increasing gestational age but non-linear increases for corresponding measures between the 2^nd^ and 3^rd^ trimester.

**Figure 2:**
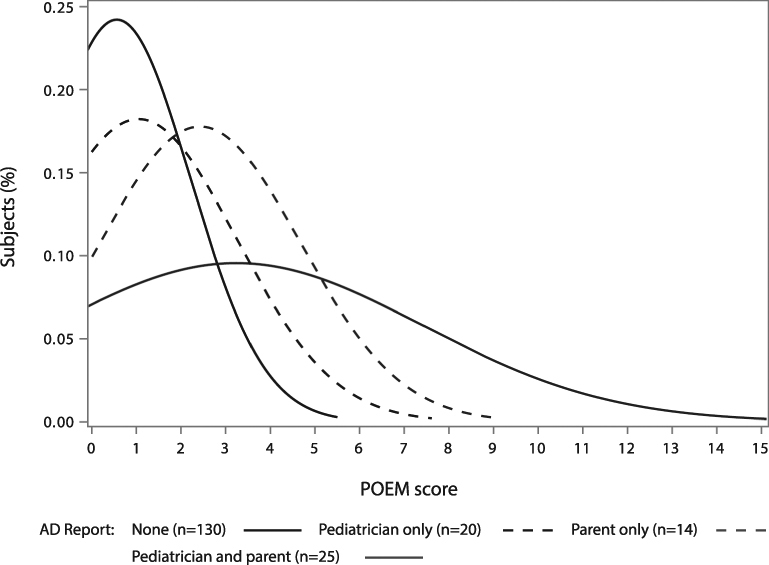
Normal density plot of Patient-Oriented Eczema Measure (POEM) scores by source of report of physician-diagnosed atopic dermatitis (AD).

Among subjects for whom POEM scores were available, AD severity was highest for those with both parent and pediatrician report (see Fig. 2).

In crude trimester-specific models, both low and high 2^nd^ trimester AC, high 2^nd^ trimester HC:AC, and lower 3^rd^ trimester HC were associated with higher AD risk (Supplement Table 1). Higher 3^rd^ trimester FL was associated with lower risk of AD.

Following adjustment for potential covariates and confounders, point estimates were similar and remained statistically significant except for 3rd trimester HC which became only marginally significant (Table 2). Point estimates did not meaningfully change following removal of subjects (n = 19) with any measurement considered a suspected outliers (data not shown). In models including additional subjects with partial but incomplete fetal growth data (Supplement Table 2), we observed similar strength of association for 2^nd^ trimester AC and HC:AC ratio while association for 3^rd^ trimester FL attenuated toward the null.

**Table 2:** Adjusted model results for association of low and high fetal anthropometric measurements with AD diagnosis by 3 years of age*.

Period	Measure	“Low” z-score ≤−1.00		“Normal”	“High” z-score ≥1.00	
		RR	(95% CI)	(reference)	RR	(95% CI)
1^st^ Trimester	Crown–rump length	1.34	(0.87; 2.05)	1.00	1.02	(0.62; 1.67)
2^nd^ Trimester	Abdominal circumference	**1.51**	**(1.01; 2.24)**	1.00	**1.83**	**(1.21; 2.76)**
	Head circumference	1.22	(0.80; 1.85)	1.00	0.89	(0.52; 1.52)
	Femur length	1.13	(0.69; 1.84)	1.00	1.06	(0.64; 1.75)
	HC to AC ratio^†^	**1.69**	**(1.16; 2.48)**	1.00	1.24	(0.79; 1.95)
	Estimated fetal weight^**^	1.35	(0.89; 2.07)	1.00	1.21	(0.75; 1.95)
3^rd^ Trimester	Abdominal circumference	1.04	(0.69; 1.59)	1.00	0.94	(0.59; 1.48)
	Head circumference	1.42	(0.98; 2.07)	1.00	1.13	(0.70; 1.80)
	Femur length	0.97	(0.62; 1.51)	1.00	**0.54**	**(0.31; 0.94)**
	HC to AC ratio^†^	0.82	(0.51; 1.31)	1.00	0.87	(0.56; 1.36)
	Estimated fetal weight^**^	1.09	(0.70; 1.71)	1.00	0.65	(0.36; 1.15)

*All models adjusted for child’s gender and maternal education, parity at delivery, smoking within year before pregnancy, and maternal report of childhood atopic dermatitis. “Low” and “high” fetal measurement categories included subjects with z-scores adjusted for gestational age at measure and gender. Results in bold were statistically significant (alpha = 0.05).

^†^Ratio equals head circumference divided by abdominal circumference.

^**^Estimated fetal weight (EFW) calculated as Log10EFW = 1.326–0.00326*AC*FL + 0.0107*HC + 0.0438*AC + 0.158*FL as described by Hadlock *et al*.^18^ where AC = abdominal circumference (cm), FL = femur length (cm), and HC = head circumference (cm).

Results from sensitivity analyses assessing fetal growth cut-point selection and restriction to subjects with complete ultrasound data yielded mostly predictable changes in point estimates. As cutoff values for “low” and “high” anthropometric categories were made less stringent by decreasing cut-points from 1.0 to 0.8 and 0.66 SD from the mean, some point estimates attenuated toward the null but in most cases remained significant (Supplement Tables 3 and 4). In models where 0.8 SD was used as a cutoff, lower 1^st^ trimester CRL was associated with increased AD risk. This association also attenuated slightly toward the null in models using a 0.66 SD cutoff and was only marginally significant. Upon inclusion of all 602 subjects with 1^st^ trimester CRL measures (e.g. excluded due to missing measurements in other trimesters) and using 0.8 and 0.6 SD cutoffs, point estimates were further attenuated but remained significant presumably due to increased power (data not shown). Point estimates were mostly similar regardless of case definition (parent- or pediatrician-reported physician diagnosed AD) but slightly weaker for lower 2^nd^ trimester AC with parent-reported AD definition (data not shown).

Among children with “normal” 2^nd^ trimester anthropometric measurements, faltering HC growth was marginally associated with higher AD risk in crude models (Table 3). When decreasing cutoff values for “low” and “high” anthropometric categories were applied, this result attenuated increasingly toward the null (Supplement Tables 5 and 6). Conversely, accelerated AC point estimates became increasingly protective reaching statistical significance in 0.66 SD cutoff models.

**Table 3:** Crude model results for association between change in 2^nd^ to 3^rd^ trimester fetal anthropometric measurements with AD diagnosis by 3 years of age among children with normal 2^nd^ trimester growth^*^.

Measure	“Normal to Low”		“Normal”	“Normal to High”	
	RR	95% CI	(reference)	RR	95% CI
Abdominal circumference	1.27	(0.73; 2.24)	1.00	0.81	(0.42; 1.58)
Head circumference	**1.59**	**(1.04; 2.43)**	1.00	1.04	(0.57; 1.90)
Femur length	1.17	(0.70; 1.93)	1.00	0.54	(0.27; 1.10)
HC to AC ratio^†^	0.79	(0.44; 1.43)	1.00	0.83	(0.45; 1.55)
Estimated fetal weight^**^	1.08	(0.59; 2.00)	1.00	0.45	(0.18; 1.16)

*“Low” and “high” fetal measurement categories included subjects with z-scores adjusted for gestational age at measure and gender ≤−1 and ≥1 respectively. Z-scores >−1 and <1 were considered “normal” growth. Results in bold were statistically significant (alpha = 0.05).

^**†**^Ratio equals head circumference/abdominal circumference.

^**^Estimated fetal weight (EFW) calculated as Log10EFW = 1.326–0.00326*AC*FL + 0.0107*HC + 0.0438*AC + 0.158*FL as described by Hadlock *et al*.^18^ where AC = abdominal circumference (cm), FL = femur length (cm), and HC = head circumference (cm).

## Discussion

Within this birth cohort study, we observed several associations for lower and higher fetal anthropometric measures throughout gestation with risk of AD diagnosis by 3 years of age. Associations observed for body length suggest that while early fetal growth impairment in the 1^st^ trimester may be associated with increased risk, longer body length in 3^rd^ trimester may be protective. However, low correlation between the two measures also suggests these associations are unlikely to be related. This is similar for observed associations of faltering 2^nd^ to 3^rd^ trimester HC and 2^nd^ trimester HC:AC. Conversely, association for lower 2^nd^ trimester HC:AC ratio is likely driven by AC. Associations observed separately for both lower and higher 2^nd^ trimester AC may be indicative of a case where divergent growth patterns result in similar increased risk. These results suggest several patterns of fetal growth possibly beginning as early as the 1^st^ trimester may influence AD risk or, more likely, may be indicative of other prenatal factors associated with AD risk.

Perhaps the most robust findings in our study are the previously unreported, and seemingly contradictory, associations for abnormal (lower and higher) 2^nd^ trimester AC. Point estimates reported in previous studies were often derived from models investigating continuous linear association between trimester-specific fetal growth measures^11,13,14^ or fetal growth over time^12,14^ and AD. Though one study reported a protective effect for larger 2^nd^ trimester HC with AD^13^, results from continuous linear analyses in other studies^11,12,14^ (none specifically including 2^nd^ trimester HC) reported mostly null results. Significant point estimates for lower and higher trimester-specific measures in our study may suggest potentially meaningful non-linear associations may have been missed in previous reports.

We attempted to further account for potential non-linear growth patterns by restricting models investigating accelerated and faltering 2^nd^ to 3^rd^ trimester growth to subjects who fell within normal range (+/−1.0, 0.8, or 0.66 SD from expected) for 2^nd^ trimester growth. Previous studies have separately reported contradictory findings in which accelerated 2^nd^ to 3^rd^ trimester AC growth was associated with both higher^12^ and lower^14^ AD risk. Point estimates for this association in our study were consistently protective but only became strong and significant when accelerated AC growth was categorized as greater than 0.66 SD higher than expected growth. Though inconsistent, our results may provide some further support to earlier findings of a protective effect of accelerated 2^nd^ to 3^rd^ trimester AC growth on AD risk. However further study with larger sample size is required to provide more clarity.

Several factors common in observational studies of this nature may have influenced our results and should be considered as potential limitations in the interpretation of our results. Most important among these are sample size, collection and interpretation of ultrasound data, and AD case definition.

To ensure comparability between point estimates across trimesters, the analysis sub-population was restricted to subjects with complete 1^st^, 2^nd^, and 3^rd^ trimester fetal growth and AD outcome data up to 3 years. Following restriction, the proportion of remaining subjects (41% of those eligible) in the analysis was comparable to one larger study^12^ and higher than other smaller studies^11,14^ with similar aims. Nevertheless, sample size may have affected our power to detect statistical significance of some associations. In particular, faltering and accelerated fetal growth models included only a relatively small proportion of subjects due to previously mentioned restrictions. We performed several analyses to investigate the effects of sample size and participation on our results. In sensitivity analyses including all subjects with at least partial trimester-specific fetal growth measurements; our sample size increased to 602 (1^st^ trimester), 445 (2^nd^ trimester), and 461 (3^rd^ trimester) subjects. Although we observed some shifts in point estimates toward the null, statistical significance was largely unaffected. These results may suggest that point estimates presented in our main results could be slightly overstated but were unlikely to have been greatly affected by selection bias. Though small sample size precluded us from adjusting faltering and accelerated growth models for potential confounders, we believe it is unlikely that adjustment for any of the factors we assessed in our analysis would have greatly affected point estimates as was the case in trimester-specific models.

Ultrasound data in our study was transcribed from paper documentation which mothers in Germany bring with them to each obstetric appointment which is then updated by their physician. Mothers were spread across 58 different obstetric practices resulting in some variation in measurement as well as documentation and potentially observer error. In some cases, documentation required calculation of HC and AC diameters. Amongst 5 practices which documented ultrasound results for 20 or more children, some individual fetal measurement results deviated from the expected mean but no practice showed consistent deviation for multiple results (data not shown). Though we could not account for potential variability within or between physician practice, one study from the UK reported inter-observer variability in interpretation of ultrasound imagery at roughly +/−4.9%, 8.8%, and 11.1% for measurement of HC, AC, and FL respectively^23^. Thus, it is possible that measurement error near cut-points defining “low” and “high” growth could have resulted in some misclassification of fetal growth. However, results from sensitivity analyses where categories were shifted from a z-score cut-point of 1.0 to 0.8 and 0.66 resulted in mostly predictable changes in estimates across this gradient. Further, individual measurements as reported in Fig. 1, were mostly within range of those reported by the Intergrowth-21 project for development of international guidelines on fetal growth^24^. Finally, model results were not noticeably different following exclusion of subjects with any single measurement considered a potential outlier. Therefore, it is unlikely our results were greatly affected by measurement error.

In contrast to previous studies which relied mostly on parent-only report of child of physician-diagnosed AD, AD cases in our study were defined using a combination of both parent and pediatrician report and, in a few cases, records from dermatological examinations. As we reported, AD severity at 3 years of age was highest for children with both a parent and pediatrician report of AD diagnosis while children with parent-only or pediatrician-only report included more mild cases yet obviously higher severity than among those with no report of AD. Thus, the AD case definition in our study may include a higher overall proportion of mildly symptomatic disease compared to other studies which may define AD using only parent report or health care data. However, when modeled separately, we observed similar point estimates for both parent-only and pediatrician-only reported outcomes. Nevertheless, as is the case in many studies of AD, some respondents or physicians may have confused AD with other forms of dermatitis. Therefore, we cannot completely rule out the possibility that some AD cases in our analysis may have been misclassified.

Several biological mechanisms have been proposed which may shed light on the potential role of fetal growth as a marker for risk of atopic disease. First trimester fetal growth restriction has been associated with several maternal demographic, lifestyle, and health-related factors^25^. Some of these factors such as maternal age and smoking are also associated with breastfeeding behavior^16^ which may thus be a proxy confounder for these maternal characteristics we accounted for when assessing associations of fetal growth with atopic outcomes. Another possibility is that fetal growth may be an indicator of other underlying factors related to fetal nutrition such as maternal diet, nutrient intake, or placental function^7^. These factors, or the resulting impact on fetal growth, may contribute to gene-environment interactions which could potentially influence skin development and/or function. Although we currently do not have data on genetic make-up of our study cohort, several gene loci have already been identified in other studies as potential study targets, the most well-known being Filaggrin^26^. In addition to genetic alteration, some evidence suggests factors associated with fetal environment may be associated with early-life programming of the inflammatory response^27^. Of note, we have recently reported association between gestational weight gain and levels of the inflammatory biomarker leptin in cord blood^28^. Finally, several studies have also suggested that postnatal growth which may be particularly associated with late-term fetal growth (e.g. “catch-up” growth) may also contribute to or explain associations with atopic dermatitis outcomes^6,7^.

Together with previous studies, our results may be interpreted as preliminary evidence suggesting that fetal growth may play a role in or be a marker for factors influencing the development of atopic disease. Most notably, our results may help clarify differential results in previous studies by demonstrating that several potential patterns of both accelerated and faltering fetal growth may be relevant. In addition, these patterns may vary depending upon developmental period of gestation. Further research may be warranted to investigate associations between non-linear patterns of fetal growth and disease particularly relatively unexplored 1^st^ trimester growth acceleration. Therefore, larger studies incorporating more precise ultrasound measurements (particularly in the 1^st^ and 2^nd^ trimester), biomarkers for atopy such as IgE levels, and inclusion of additional factors which may affect fetal growth and possibly shed light on possible gene-environment interactions or immune-related fetal programming during gestation may be warranted. Finally, the generalizability of these results should be explored across geographically and culturally different study populations which may differ in both fetal growth and atopic outcome characteristics.

## Electronic supplementary material

Supplementary Information
