# Supplementary material for: Fetal growth and incidence of atopic dermatitis in early childhood: Results of the Ulm SPATZ Health Study
Source: Sci Rep. 2018 May 23;8:8041. doi: 10.1038/s41598-018-26440-2 (PMC5966404; doi:10.1038/s41598-018-26440-2)
Supplement: Supplementary file 1 — Supplementary Information [file 41598_2018_26440_MOESM1_ESM.docx]

**Online data supplement: Fetal growth and incidence of atopic dermatitis in early childhood: Results of the Ulm SPATZ Health Study**

Chad A. Logan ^1^, Johannes M. Weiss ^2^, Frank Reister ^3^, Dietrich Rothenbacher ^1^, Jon Genuneit ^1,4^

^1^ Institute of Epidemiology and Medical Biometry, Ulm University, Ulm, Germany

^2^ Department of Dermatology and Allergic Diseases, University Medical Center Ulm, Ulm, Germany

^3^ Department of Gynecology and Obstetrics, University Medical Center Ulm, Ulm, Germany

^4^ Member of ‘In-FLAME’ the International Inflammation Network, World Universities Network (WUN)

Address correspondence to: PD Dr. med. Jon Genuneit, MSc, Institute of Epidemiology and Medical Biometry, Ulm University, Helmholtzstr. 22, D-89081 Ulm, Germany, [jon.genuneit@uni-ulm.de], phone: 0049 731 500 31067, fax: 0049 731 5012 31067

**Supplement Table 1: Crude model results for association of low and high fetal anthropometric measurements with AD diagnosis categorized by 3 years of age***

|  |  | **“Low” z-score ≤ -1.00** | | **“Normal”** | **“High” z-score ≥ 1.00** | |
| --- | --- | --- | --- | --- | --- | --- |
| **Period** | **Measure** | **RR** | **(95%CI)** | **(reference)** | **RR** | **(95%CI)** |
| 1^st^ Trimester | Crown–rump length | 1.38 | (0.91; 2.10) | 1.00 | 0.99 | (0.60; 1.64) |
| 2^nd^ Trimester | Abdominal circumference | **1.53** | **(1.03; 2.28)** | 1.00 | **1.78** | **(1.19; 2.66)** |
|  | Head circumference | 1.17 | (0.76; 1.82) | 1.00 | 0.84 | (0.49; 1.45) |
|  | Femur length | 1.13 | (0.69; 1.83) | 1.00 | 1.05 | (0.64; 1.72) |
|  | HC to AC ratio† | **1.68** | **(1.15; 2.46)** | 1.00 | 1.26 | (0.80; 1.97) |
|  | Estimated fetal weight**^**^** | 1.29 | (0.86; 1.95) | 1.00 | 1.10 | (0.69; 1.76) |
| 3^rd^ Trimester | Abdominal circumference | 1.09 | (0.71; 1.67) | 1.00 | 0.92 | (0.58; 1.46) |
|  | Head circumference | **1.46** | **(1.00; 2.13)** | 1.00 | 1.11 | (0.69; 1.80) |
|  | Femur length | 1.02 | (0.66; 1.57) | 1.00 | **0.56** | **(0.32; 0.98)** |
|  | HC to AC ratio† | 0.83 | (0.51; 1.33) | 1.00 | 0.91 | (0.58; 1.44) |
|  | Estimated fetal weight**^**^** | 1.15 | (0.74; 1.78) | 1.00 | 0.65 | (0.36; 1.17) |

^*^ “Low” and “high” fetal measurement categories included subjects with z-scores adjusted for gestational age at measure and gender.

^†^ Ratio equals head circumference divided by abdominal circumference

^**^ Estimated fetal weight (EFW) calculated as Log10EFW = 1.326-0.00326*AC*FL+0.0107*HC+0.0438*AC+0.158*FL as described by Hadlock et al^1^ where AC = abdominal circumference (cm), FL = femur length (cm), and HC = head circumference (cm)

**Supplement Table 2: Adjusted model results for association of low and high fetal anthropometric measurements with AD diagnosis by 3 years of age including subjects with all or only partial ultrasound data***

|  |  | **“Low” z-score ≤ -1.00** | | **“Normal”** | **“High” z-score ≥ 1.00** | |
| --- | --- | --- | --- | --- | --- | --- |
| **Period** | **Measure** | **RR** | **(95%CI)** | **(reference)** | **RR** | **(95%CI)** |
| 1^st^ Trimester | Crown–rump length | 1.18 | (0.83; 1.67) | 1.00 | 0.88 | (0.58; 1.35) |
| 2^nd^ Trimester | Abdominal circumference | **1.45** | **(1.01; 2.10)** | 1.00 | **1.54** | **(1.03; 2.32)** |
|  | Head circumference | 1.30 | (0.92; 1.85) | 1.00 | 0.98 | (0.65; 1.47) |
|  | Femur length | 1.24 | (0.86; 1.80) | 1.00 | 0.99 | (0.66; 1.49) |
|  | HC to AC ratio† | **1.56** | **(1.07; 2.26)** | 1.00 | 1.30 | (0.85; 1.97) |
|  | Estimated fetal weight**^**^** | 1.34 | (0.90; 1.99) | 1.00 | 1.08 | (0.68; 1.69) |
| 3^rd^ Trimester | Abdominal circumference | 0.94 | (0.63; 1.39) | 1.00 | 0.85 | (0.55; 1.31) |
|  | Head circumference | 1.34 | (0.97; 1.86) | 1.00 | 0.88 | (0.57; 1.36) |
|  | Femur length | 1.01 | (0.70; 1.46) | 1.00 | 0.73 | (0.48; 1.11) |
|  | HC to AC ratio† | 0.92 | (0.60; 1.41) | 1.00 | 0.76 | (0.48; 1.19) |
|  | Estimated fetal weight**^**^** | 1.09 | (0.71; 1.67) | 1.00 | 0.72 | (0.43; 1.19) |

^*^ All models adjusted for child’s gender and maternal education, parity at delivery, smoking within year before pregnancy, and maternal report of childhood atopic dermatitis. “Low” and “high” fetal measurement categories included subjects with z-scores adjusted for gestational age at measure and gender. Results in bold were statistically significant (alpha = 0.05). Model subject counts 1st trimester n=602, 2nd trimester n=445, and 3rd trimester n=461.

^†^ Ratio equals head circumference divided by abdominal circumference

^**^ Estimated fetal weight (EFW) calculated as Log10EFW = 1.326-0.00326*AC*FL+0.0107*HC+0.0438*AC+0.158*FL as described by Hadlock et al^1^ where AC = abdominal circumference (cm), FL = femur length (cm), and HC = head circumference (cm)

**Supplement Table 3: Adjusted model results for association of low and high fetal anthropometric measurements with AD diagnosis categorized using z-score cut-points at +/-0.80SD with AD diagnosis by 3 years of age***

|  |  | **“Low” z-score ≤ -0.80** | | **“Normal”** | **“High” z-score ≥ 0.80** | |
| --- | --- | --- | --- | --- | --- | --- |
| **Period** | **Measure** | **RR** | **(95%CI)** | **(reference)** | **RR** | **(95%CI)** |
| 1^st^ Trimester | Crown–rump length | **1.51** | **(1.03; 2.22)** | 1.00 | 1.29 | (0.85; 1.96) |
| 2^nd^ Trimester | Abdominal circumference | **1.62** | **(1.12; 2.32)** | 1.00 | **1.66** | **(1.11; 2.48)** |
|  | Head circumference | 1.21 | (0.84; 1.75) | 1.00 | 0.95 | (0.60; 1.49) |
|  | Femur length | 1.06 | (0.67; 1.65) | 1.00 | 0.96 | (0.61; 1.52) |
|  | HC to AC ratio† | **1.70** | **(1.18; 2.44)** | 1.00 | 1.42 | (0.95; 2.12) |
|  | Estimated fetal weight**^**^** | 1.31 | (0.87; 1.97) | 1.00 | 1.28 | (0.85; 1.91) |
| 3^rd^ Trimester | Abdominal circumference | 1.00 | (0.69; 1.45) | 1.00 | 0.78 | (0.50; 1.21) |
|  | Head circumference | **1.43** | **(1.00; 2.04)** | 1.00 | 1.05 | (0.68; 1.62) |
|  | Femur length | 0.91 | (0.61; 1.36) | 1.00 | **0.57** | **(0.35; 0.93)** |
|  | HC to AC ratio† | 0.79 | (0.52; 1.20) | 1.00 | 0.76 | (0.50; 1.15) |
|  | Estimated fetal weight**^**^** | 1.08 | (0.74; 1.59) | 1.00 | 0.77 | (0.47; 1.24) |

^*^ All models adjusted for child’s gender and maternal education, parity at delivery, smoking within year before pregnancy, and maternal report of childhood atopic dermatitis. “Low” and “high” fetal measurement categories included subjects with z-scores adjusted for gestational age at measure and gender. Results in bold were statistically significant (alpha = 0.05).

^†^ Ratio equals head circumference divided by abdominal circumference

^**^ Estimated fetal weight (EFW) calculated as Log10EFW = 1.326-0.00326*AC*FL+0.0107*HC+0.0438*AC+0.158*FL as described by Hadlock et al^1^ where AC = abdominal circumference (cm), FL = femur length (cm), and HC = head circumference (cm)

**Supplement Table 4: Adjusted model results for association of low and high fetal anthropometric measurements with AD diagnosis categorized using z-score cut-points at +/-0.66SD with AD diagnosis by 3 years of age***

|  |  | **“Low” z-score ≤ -0.66** | | **“Normal”** | **“High” z-score ≥ 0.66** | |
| --- | --- | --- | --- | --- | --- | --- |
| **Period** | **Measure** | **RR** | **(95%CI)** | **(reference)** | **RR** | **(95%CI)** |
| 1^st^ Trimester | Crown–rump length | 1.44 | (0.99; 2.09) | 1.00 | 1.22 | (0.81; 1.84) |
| 2^nd^ Trimester | Abdominal circumference | 1.38 | (0.96; 2.00) | 1.00 | **1.56** | **(1.06; 2.30)** |
|  | Head circumference | 1.34 | (0.94; 1.91) | 1.00 | 1.02 | (0.66; 1.55) |
|  | Femur length | 1.08 | (0.71; 1.63) | 1.00 | 1.10 | (0.74; 1.63) |
|  | HC to AC ratio† | **1.78** | **(1.24; 2.56)** | 1.00 | **1.57** | **(1.06; 2.33)** |
|  | Estimated fetal weight**^**^** | 1.23 | (0.82; 1.83) | 1.00 | 1.28 | (0.86; 1.90) |
| 3^rd^ Trimester | Abdominal circumference | 0.93 | (0.64; 1.35) | 1.00 | 0.77 | (0.51; 1.15) |
|  | Head circumference | 1.27 | (0.89; 1.80) | 1.00 | 0.98 | (0.64; 1.48) |
|  | Femur length | 0.99 | (0.68; 1.44) | 1.00 | **0.62** | **(0.39; 0.98)** |
|  | HC to AC ratio† | 0.90 | (0.61; 1.32) | 1.00 | 0.80 | (0.54; 1.20) |
|  | Estimated fetal weight**^**^** | 1.27 | (0.89; 1.82) | 1.00 | 0.85 | (0.55; 1.32) |

^*^ All models adjusted for child’s gender and maternal education, parity at delivery, smoking within year before pregnancy, and maternal report of childhood atopic dermatitis. “Low” and “high” fetal measurement categories included subjects with z-scores adjusted for gestational age at measure and gender. Results in bold were statistically significant (alpha = 0.05).

^†^ Ratio equals head circumference divided by abdominal circumference

^**^ Estimated fetal weight (EFW) calculated as Log10EFW = 1.326-0.00326*AC*FL+0.0107*HC+0.0438*AC+0.158*FL as described by Hadlock et al^1^ where AC = abdominal circumference (cm), FL = femur length (cm), and HC = head circumference (cm)

**Supplement Table 5: Crude model results for association between change in 2^nd^ to 3^rd^ trimester fetal anthropometric measurements categorized using z-score cut-points at +/-0.80SD and AD diagnosis by 3 years of age among children with normal 2^nd^ trimester growth^*^**

|  | **“Normal to Low”** | | **“Normal”** | **“Normal to High”** | |
| --- | --- | --- | --- | --- | --- |
| **Measure** | **RR** | **95%CI** | **(reference)** | **RR** | **95%CI** |
| Abdominal circumference | 1.34 | (0.80; 2.24) | 1.00 | 0.64 | (0.32; 1.28) |
| Head circumference | 1.31 | (0.81; 2.13) | 1.00 | 0.90 | (0.49; 1.63) |
| Femur length | 1.05 | (0.64; 1.70) | 1.00 | 0.54 | (0.28; 1.01) |
| HC to AC ratio† | 0.92 | (0.51; 1.65) | 1.00 | 0.97 | (0.53; 1.78) |
| Estimated fetal weight**^**^** | 1.28 | (0.74; 2.19) | 1.00 | 0.62 | (0.29; 1.34) |

^*^ “Low” and “high” fetal measurement categories included subjects with z-scores adjusted for gestational age at measure and gender ≤ -0.80 and ≥ 0.80 respectively. Z-scores >-1 and <1 were considered “normal” growth. Results in bold were statistically significant (alpha = 0.05).

**^†^** Ratio equals head circumference divided by abdominal circumference

^**^ Estimated fetal weight (EFW) calculated as Log10EFW = 1.326-0.00326*AC*FL+0.0107*HC+0.0438*AC+0.158*FL as described by Hadlock^1^ et al where AC = abdominal circumference (cm), FL = femur length (cm), and HC = head circumference (cm)

**Supplement Table 6: Crude model results for association between change in 2^nd^ to 3^rd^ trimester fetal anthropometric measurements categorized using z-score cut-points at +/-0.66SD with AD diagnosis by 3 years of age among children with normal 2^nd^ trimester growth^*^**

|  | **“Normal to Low”** | | **“Normal”** | **“Normal to High”** | |
| --- | --- | --- | --- | --- | --- |
| **Measure** | **RR** | **95%CI** | **(reference)** | **RR** | **95%CI** |
| Abdominal circumference | 1.14 | (0.69; 1.89) | 1.00 | **0.38** | **(0.17; 0.86)** |
| Head circumference | 1.16 | (0.69; 1.94) | 1.00 | 0.86 | (0.47; 1.58) |
| Femur length | 1.13 | (0.68; 1.86) | 1.00 | 0.56 | (0.28; 1.12) |
| HC to AC ratio† | 1.02 | (0.55; 1.89) | 1.00 | 0.89 | (0.44; 1.80) |
| Estimated fetal weight**^**^** | 1.48 | (0.89; 2.46) | 1.00 | 0.78 | (0.41; 1.50) |

^*^ “Low” and “high” fetal measurement categories included subjects with z-scores adjusted for gestational age at measure and gender ≤ -0.80 and ≥ 0.80 respectively. Z-scores >-1 and <1 were considered “normal” growth. Results in bold were statistically significant (alpha = 0.05).

**^†^** Ratio equals head circumference divided by abdominal circumference

^**^ Estimated fetal weight (EFW) calculated as Log10EFW = 1.326-0.00326*AC*FL+0.0107*HC+0.0438*AC+0.158*FL as described by Hadlock^1^ et al where AC = abdominal circumference (cm), FL = femur length (cm), and HC = head circumference (cm)

**Supplement Figure 1: Boxplot comparisons for measured and calculated 2^nd^ and 3^rd^ trimester head and abdominal circumferences***

**
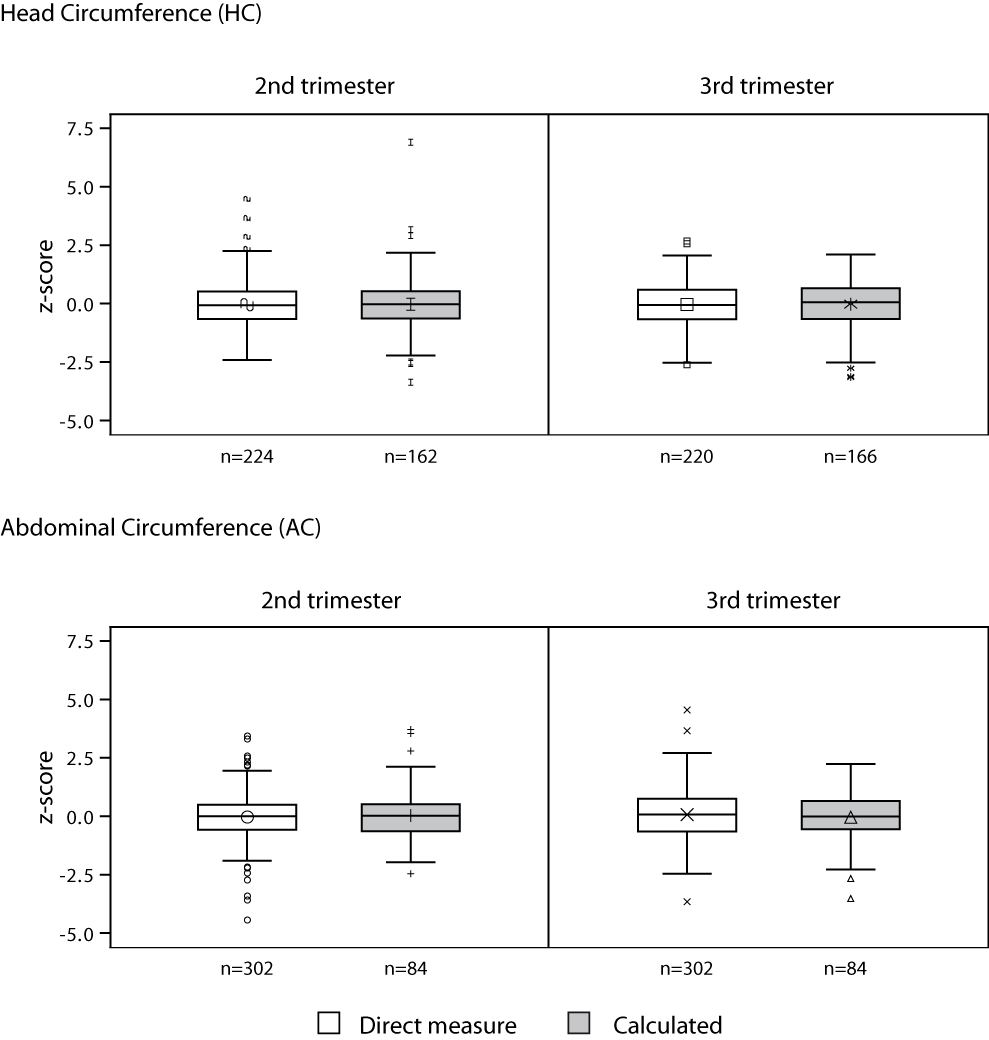
**

* Kruskal-Wallis sum rank test p-values indicated no significant difference in distribution between any measured and calculated circumference pairing (2^nd^ trimester HC p = 0.74; 3^rd^ trimester HC p = 0.42; 2^nd^ trimester AC p = 0.91; 3^rd^ trimester AC p = 0.52)

**REFERENCES**

1. Hadlock, F. P., Harrist, R. B., Carpenter, R. J., Deter, R. L. & Park, S. K. Sonographic estimation of fetal weight. The value of femur length in addition to head and abdomen measurements. *Radiology* **150,** 535–540 (1984).
